# An Unusual Case of an Extra-salivary Subcutaneous Multifocal Pleomorphic Salivary Adenoma

**DOI:** 10.7759/cureus.99723

**Published:** 2025-12-20

**Authors:** Conor Gordon, Shailendra Walunj, Gordon Hutchins, Mohammed Anabtawi

**Affiliations:** 1 Oral and Maxillofacial Surgery, Leeds General Infirmary, Leeds, GBR; 2 Radiology, Pinderfields Hospital, Wakefield, GBR; 3 Histopathology, Leeds General Infirmary, Leeds, GBR

**Keywords:** head and neck pathologies, pleomorphic adenomas, pleomorphic salivary adenoma, salivary gland tumor, subcutaneous nodules

## Abstract

A 36-year-old woman was referred to the Oral and Maxillofacial Surgery clinic at Pinderfields Hospital, Wakefield, with multiple subcutaneous swellings on the right side of the face, overlying the parotid region and extending below the mandibular border. Magnetic resonance imaging (MRI) revealed several macrocystic subcutaneous lesions, and ultrasound-guided fine-needle aspiration confirmed a diagnosis of pleomorphic salivary adenoma (PSA). The lesions were excised via a modified Blair incision and were found to be superficial to the parotid capsule. Histopathological evaluation showed myxoid-rich PSAs without any associated salivary gland tissue, confirming an extrasalivary origin.

This case illustrates a rare presentation of multifocal, subcutaneous PSAs occurring independently of the parotid gland. The patient remains disease-free and continues under routine surveillance for potential recurrence.

## Introduction

Pleomorphic salivary adenomas (PSA) are the most common benign salivary neoplasms [1]. They make up approximately 65% of all salivary gland tumours [2]. These lesions are characterised histologically by their diverse cellular composition, which includes epithelial, myoepithelial, and stromal elements; hence, the designation “pleomorphic”. Clinically, PSAs typically manifest as slow-growing, well-circumscribed, painless solitary masses within salivary gland tissue.

Reported incidences vary, but the most common site in which PSAs are located is the parotid gland [1,3]. PSAs can also originate in the submandibular and sublingual glands as well as the numerous minor salivary glands located throughout the oral cavity such as the soft palate, lips and buccal mucosa.

Extra-salivary pleomorphic adenomas are rare and may occur in ectopic salivary gland tissue or at accessory sites. These unusual presentations are thought to arise from heterotopic salivary gland tissue or embryologic remnants deposited along the developmental fusion plane.

We present a case that demonstrates an unusual presentation of a multifocal extra-salivary subcutaneous PSA in a 36-year-old female patient with no prior surgical history involving the salivary glands or adjacent anatomical structures. The patient presented with multiple clinically palpable lesions situated over the right parotid gland, the right angle of the mandible and the superior aspect of the right sternomastoid muscle.

## Case presentation

**Figure 1:**
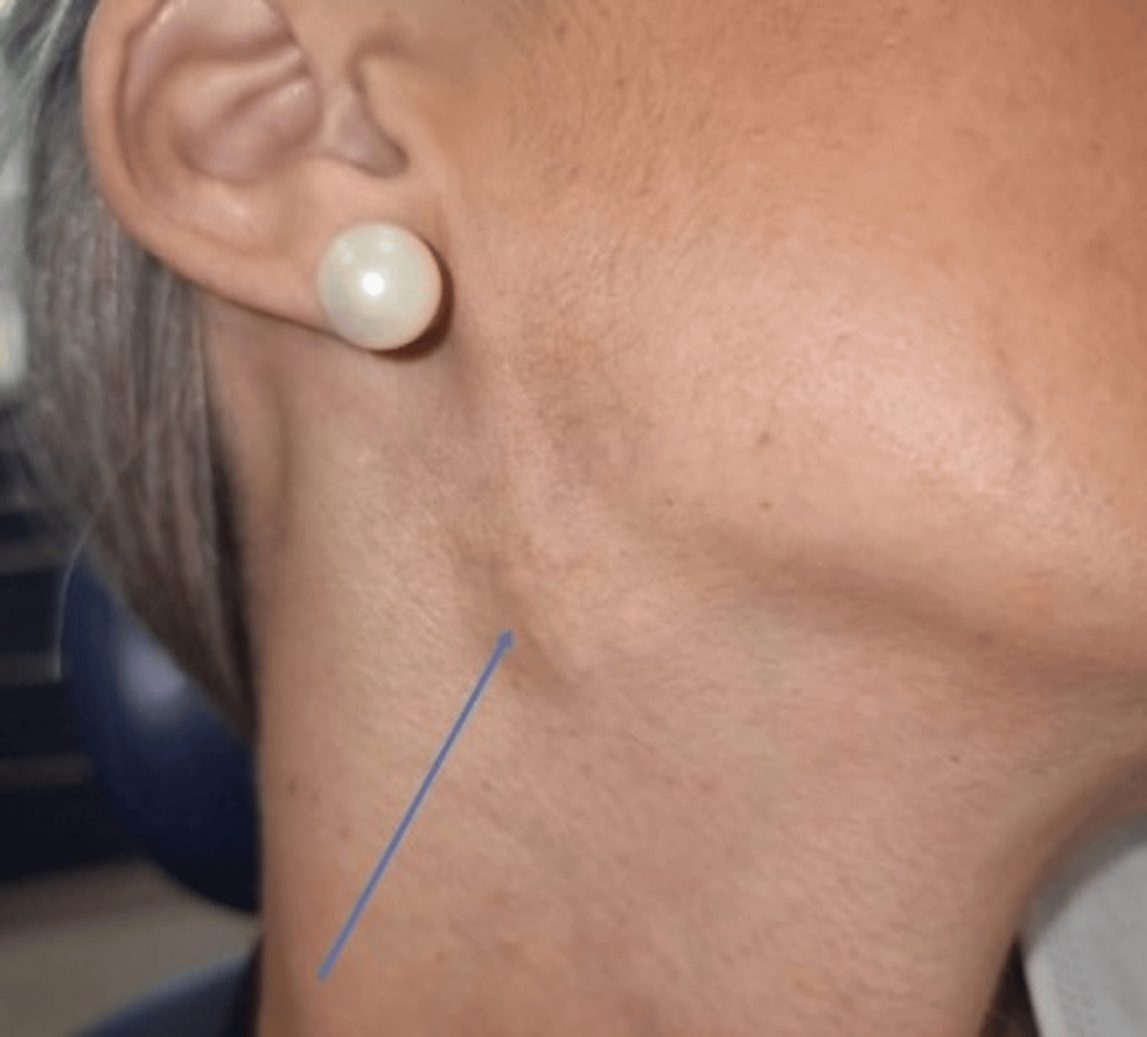
Clinical photo of the lesion below the right angle of the mandible

A 36-year-old female was referred to the oral and maxillofacial surgery (OMFS) clinic at Pinderfields Hospital, Wakefield. The patient was referred due to subcutaneous lumps on the right side of her face extending below the lower border of the mandible (Figure 1). The patient had no history of parotid gland surgery, but as a teenager, had a subcutaneous nodule removed under local anaesthesia (LA) in the right mastoid region at a local clinic in Greece. Unfortunately, no further medical records were available regarding this. The patient had previously undergone a laparoscopic cholecystectomy for symptomatic gallstones but was otherwise fit and well and took no regular medications. She had noticed this area develop over a number of weeks preceding the referral. They were not causing any significant pain or discomfort, and she reported no facial muscle weakness.

**Figure 2:**
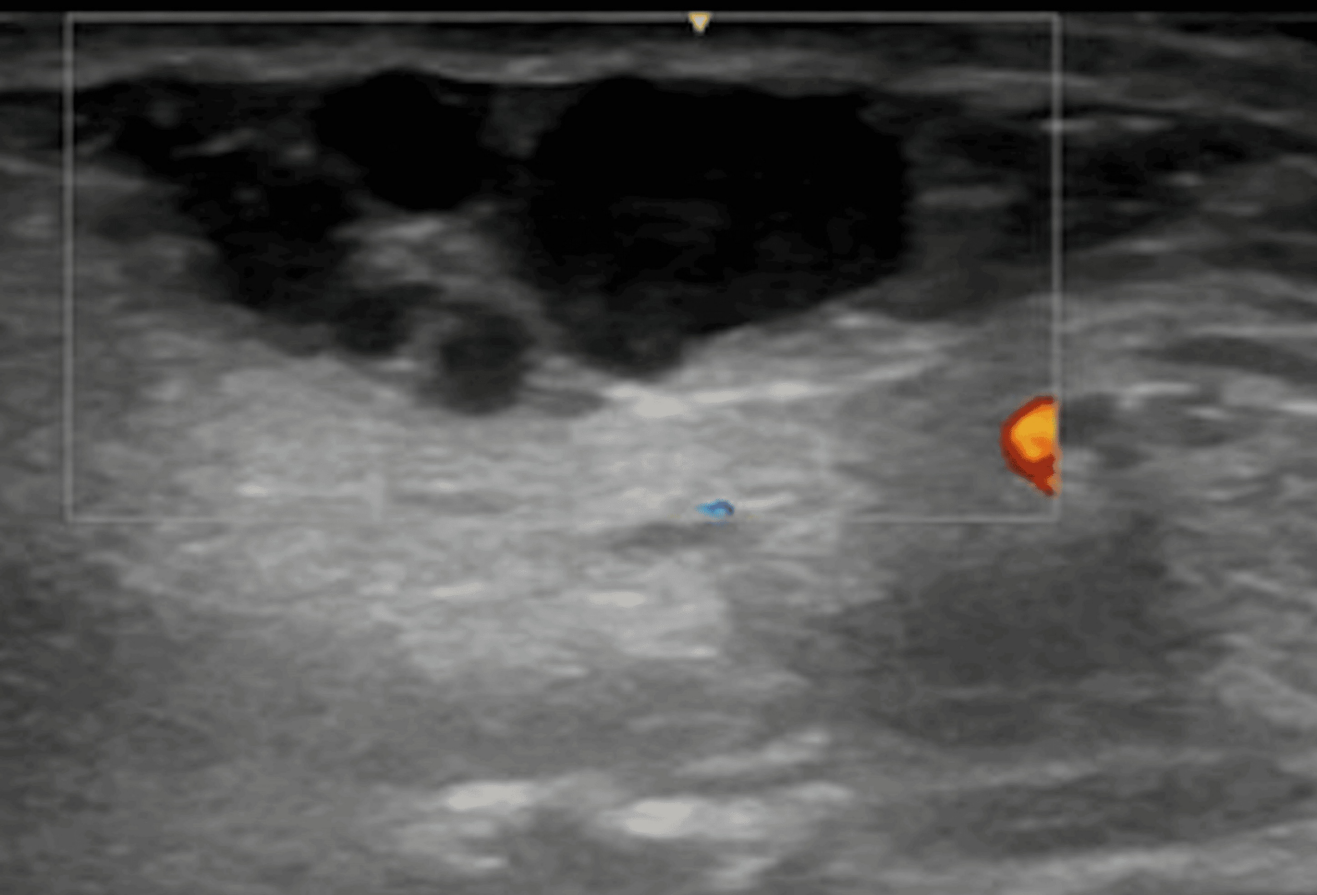
Ultrasound scan demonstrating lesions

The patient was referred for an ultrasound scan in the community by her general practitioner before referral, which demonstrated several hypoechoic avascular lesions in the subcutaneous layer (Figure 2). These were distinctly separate from the underlying parotid gland. The largest measured 27 x 9mm. The patient was assessed in the OMFS department, and multiple, firm, painless lesions were palpable subcutaneously overlying the right parotid gland and extending below the lower border of the mandible to level 2A of the neck. These lesions were not obviously tethered to deeper structures. No facial nerve weakness or parapharyngeal swelling was clinically present.

**Figure 3:**
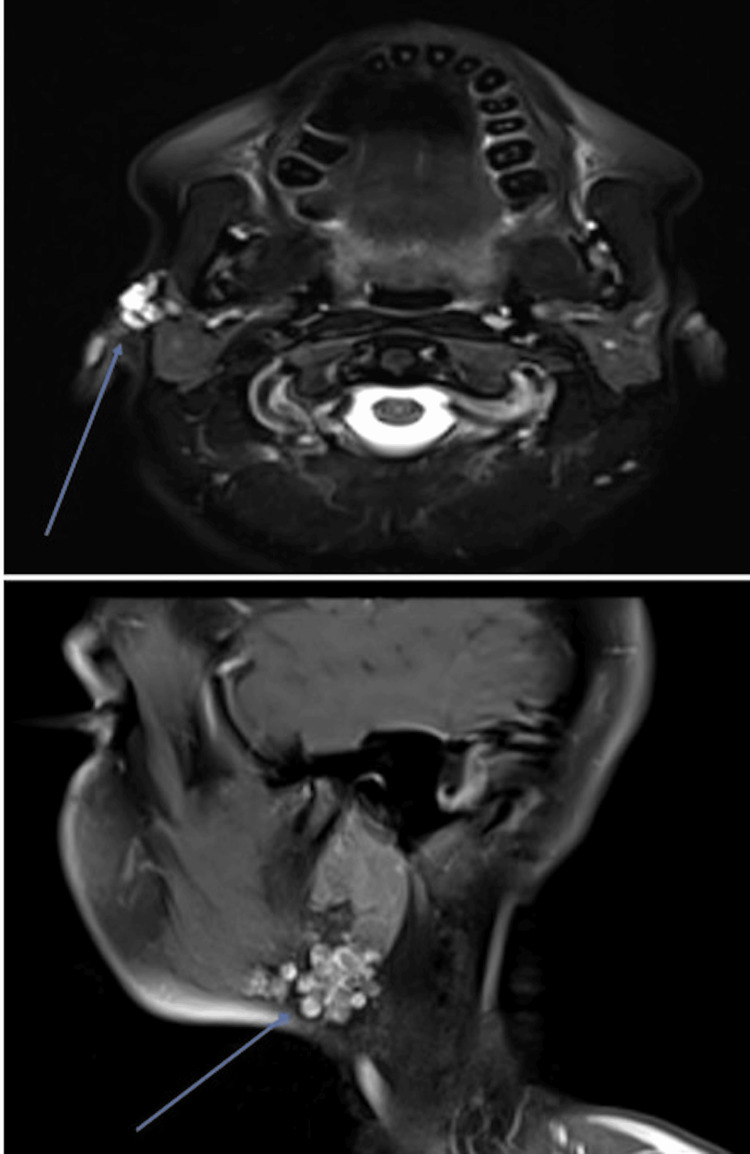
Axial and sagittal MRI images demonstrating the multifocal subcutaneous lesions

A magnetic resonance imaging (MRI) of the area was requested to aid diagnosis and definitive treatment planning. This revealed minimally enhancing, multifocal macro cystic lesions located subcutaneously, extending from the right pre-auricular region to below the inferior border of the mandible (Figure 3). These lesions were situated superficial to the parotid capsule without evidence of capsular breach. A transpatial lesion, such as a lymphovenous malformation, was considered a close imaging differential diagnosis. Ultrasound-guided fine needle aspiration (FNA) was then requested, and cytology subsequently confirmed PSA. The patient was discussed at the local head and neck multidisciplinary team (MDT) meeting. Multiple treatment modalities were considered, but the decision was to offer surgical removal of the lesions, and this was carried out under general anaesthesia. The access was via a right-sided modified Blair approach. The flap was raised in a subcutaneous plane, and intraoperatively, the nodules were located overlying the sternocleidomastoid muscle and extended up to 5 cm below the lower border of the mandible. The superior lesions were above the level of the parotid capsule, and there was no clinically macroscopic evidence of ectopic salivary gland tissue. Care had to be taken when removing the lesions to protect the subdermal plexus supplying the overlying skin paddle so as to avoid necrosis.

**Figure 4:**
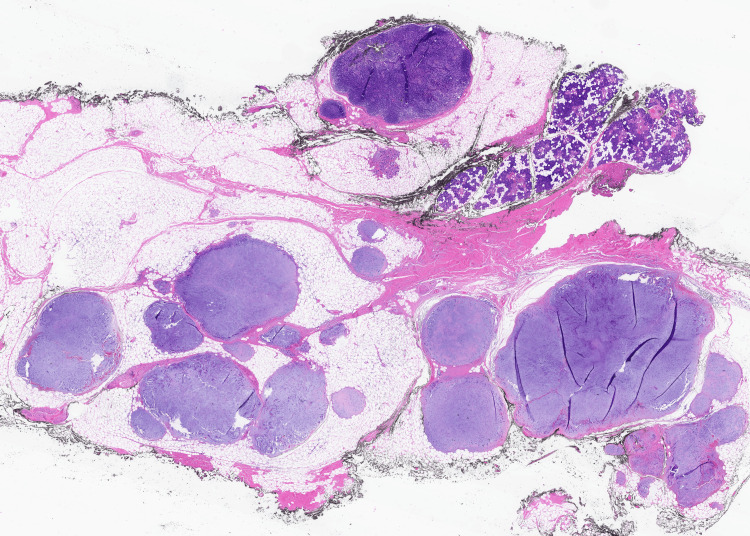
Histological slide demonstrating PSA PSA: pleomorphic salivary adenoma

**Figure 5:**
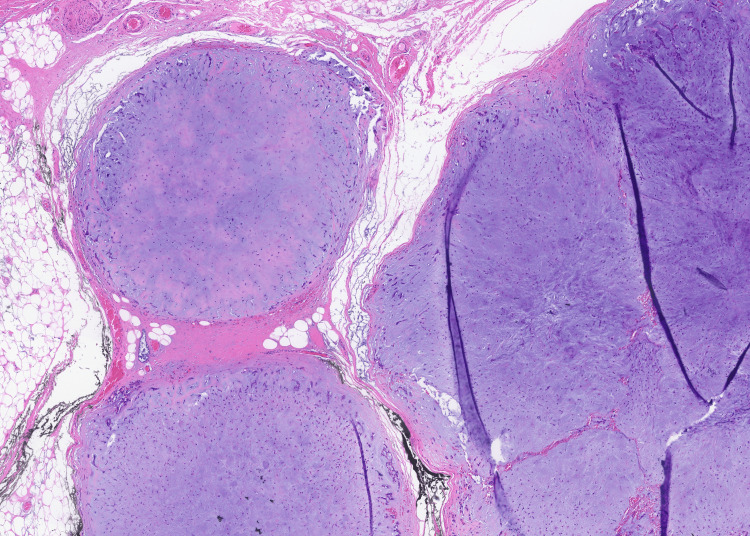
Histological slide demonstrating PSA PSA: pleomorphic salivary adenoma

Histological analysis of the specimen (Figures 4-5) subsequently confirmed the lesions to be myxoid-rich PSAs. Ribonucleic acid (RNA) extracted from formalin-fixed paraffin-embedded tumour tissue was screened for gene fusion transcripts associated with a range of cancer types using the Illumina TruSight RNA Fusion Panel (Illumina, Inc., San Diego, CA, US). HMGA2::WIF1 fusion was detected, which is consistent with pleomorphic salivary adenoma, although this has also been detected in other salivary gland tumours [4,5]. 

**Figure 6:**
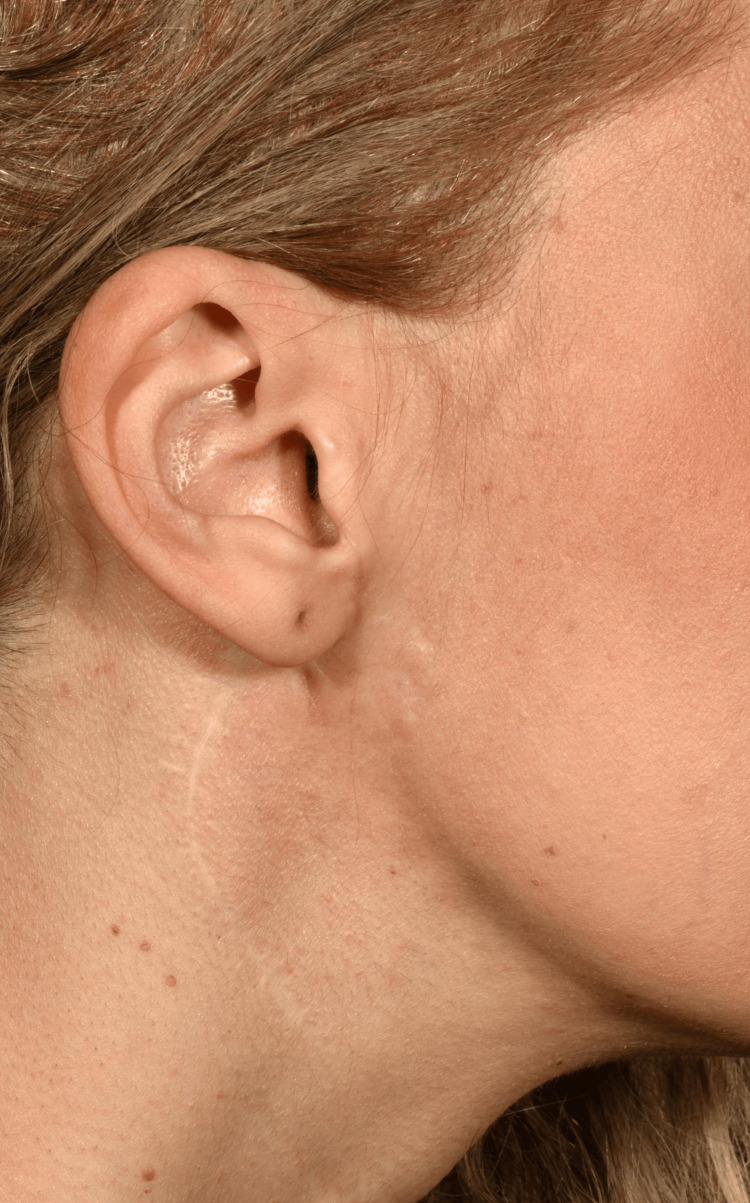
Eighteen months post-operative

At clinical follow-up, the surgical site had healed well with no patient concerns (Figure 6). The scar had camouflaged well with the surrounding skin. The patient remains under close clinical follow-up with no signs of local recurrence 18 months post-operatively.

## Discussion

Salivary gland tumours are rare, with an incidence of approximately 2.5-3 cases per 100,000 individuals [6]. Pleomorphic salivary adenomas (PSAs) are the most common benign salivary gland neoplasm. They typically present as slow-growing, solitary, painless masses. Owing to their complex morphological features, PSAs arise from both epithelial and myoepithelial cells. They most frequently involve the parotid gland but can also occur in other major salivary glands and in minor salivary gland sites such as the palate, buccal mucosa, and lip [7-10].

Multifocal PSAs have been reported in the literature, and these multifocal lesions are usually confined to salivary gland tissue. Tumour spillage during prior surgery, seeding during biopsy and pseudopodial extensions from the tumour capsule have all been implicated as potential mechanisms for multifocal disease.

Extrasalivary PSAs are uncommon. Isolated case reports describe PSAs arising within subcutaneous tissues, including the nasolabial fold, eyelid, and preauricular region [11,12]. Such lesions may originate from ectopic salivary gland tissue; however, no salivary gland tissue was identified in our histological specimens. In this case, the lesions were detectable on imaging and clinical examination prior to diagnostic sampling, and no additional salivary gland abnormalities were identified on MRI or ultrasound.

## Conclusions

This case highlights the unusual presentation of multifocal PSAs arising within the subcutaneous tissues overlying, but distinct from, the right parotid gland. This represents a rare extrasalivary manifestation, and clinicians should remain aware of this possibility when evaluating atypical head and neck masses. The patient is currently disease-free and remains under clinical follow-up to monitor for recurrence.
